# Correction: Synthetization and characterization of SnCaAl_2_O_3_ nanocomposite and using as a superior adsorbent for Pb, Zn, and Cd ions in polluted water

**DOI:** 10.1371/journal.pone.0306279

**Published:** 2024-06-25

**Authors:** The PLOS ONE Staff

In the Funding statement, the grant number from the funder Deanship of Scientific Research at Umm Al-Qura University is listed incorrectly. The correct grant number is: 22UQU4280446DSR01.

The ORCID iD is missing for the second author. Please see the author’s respective ORCID iD here:

- Author Moustafa Gamal Snousy’s ORCID iD is: 0000-0002-1485-1098 (https://orcid.org/0000-0002-1485-1098).

Multiple chemical compounds and acronyms appear incorrectly throughout the article. Chemical compounds “Al2O3”, “SnCaAl2O3”, and “Al(NO3)3 are incorrect. They should read “Al_2_O_3_”, “SnCaAl_2_O_3_”, and “Al(NO_3_)_3_”, respectively. The acronyms C.N.P., N.P., U.S.A., Al–O.H., C.C.P., T.G., D.T.A., T.E.M., S.E.M., B.E.T., B.J.H., N.P.s, P.F.O., T.E.M., S.E.M., and B.E.T. appear incorrectly throughout the article. The correct acronyms are CNP, NP, USA, Al–OH, CCP, TG, DTA, TEM, SEM, BET, BJH, NPs, PFO, TEM, SEM, and BET, respectively.

In the Kinetics models subsection of Adsorption kinetics and isotherm models under Results and Discussion, there is an error in the second sentence of the second paragraph. The correct sentence is: The straight-line emerges through plotting log(*qe − qt*) against t. Although, K_1_ and the theoretical *qe* may be derived from slope and intercept (S1 Fig).

In the PDF, there is an error in the first sentence of the Heavy metals adsorption by SnCaAl_2_O_3_ core-shell nanoparticles subsection of the Materials and Methods. The correct sentence is: The sorption characteristics of the as-synthesized SnCaAl_2_O_3_ CNPs were determined using the technique of limited volume at room temperature (20 ± 2˚C).

The reference links to references 2, 8, 10, 15, 19, 21, 23, 24, 27, 33, 34, 35, 36, 37, 44, 45, 51, 52, 63, 71, 77, 78, 80, 83, 86, 87 should have not been indicated.

The publisher apologizes for the errors.
