# Supplementary material for: Synthetization and characterization of SnCaAl2O3 nanocomposite and using as a superior adsorbent for Pb, Zn, and Cd ions in polluted water
Source: PLoS One. 2022 Nov 3;17(11):e0276888. doi: 10.1371/journal.pone.0276888 (PMC9632833; doi:10.1371/journal.pone.0276888)
Supplement: S1 Fig — (DOCX) [file pone.0276888.s001.docx]

**Supplementary (S1)**

**Synthetization and Characterization of SnCaAl_2_O_3_ Nanocomposite as a Superior Adsorbent for Pb, Zn, and Cd Ions in Polluted Water**

Ali Sayqal^1^, Moustafa Gamal Snousy^2^, Mahmoud F. Mubarak^3^, Ahmed H. Ragab^4^, Atef Mohamed Gad Mohamed^5^*, and Abeer El Shahawy ^6^*

^1^ Department of Chemistry, Faculty of Applied Science, Umm-Al-Qura University, Makkah, Saudi- Arabia; [aasayqal@uqu.edu.sa](mailto:aasayqal@uqu.edu.sa)

^2^ Egyptian Petroleum Sector, Petrotrade Co., Cairo P.O. Box 11371, Egypt; [moustafa_gamal93@yahoo.com](mailto:moustafa_gamal93@yahoo.com)

^3^ Petroleum Applications Department, Egyptian Petroleum Research Institute (EPRI), Cairo P.O. Box 11727, Egypt; [fathy8753@epri.sci.eg](mailto:fathy8753@epri.sci.eg)

^4^ Department of Chemistry, Faculty of Science, King Khalid University, Abha 62224, Saudi Arabia; [ahrejab@kku.edu.sa](mailto:ahrejab@kku.edu.sa)

^5^* Assiut & New Valley Company for Water and Wastewater, Assiut P.O. Box 71516, Egypt; [atefgad98@yahoo.com](mailto:atefgad98@yahoo.com)

^6^* Department of Civil Engineering, Faculty of Engineering, Suez Canal University, Ismailia P.O. Box 41522, Egypt; [abeer_shahawi@eng.suez.edu.eg](mailto:abeer_shahawi@eng.suez.edu.eg)

*****Correspondence: [abeer_shahawi@eng.suez.edu.eg](mailto:abeer_shahawi@eng.suez.edu.eg),

[atefgad98@yahoo.com](mailto:atefgad98@yahoo.com)

|  |  |
| --- | --- |
| (a) | (b) |
|  |  |
| (c) | (d) |
|  |  |
| (e) | (f) |

Figure S1**.** Adsorption kinetics of (a,b) Zn^+2^, (c,d) Pb^+2^, and (e,f) Cd^+2^ adsorption on SnCaAl_2_O_3_ nanoadsorbent.
