# Supplementary material for: Synthetization and characterization of SnCaAl2O3 nanocomposite and using as a superior adsorbent for Pb, Zn, and Cd ions in polluted water
Source: PLoS One. 2022 Nov 3;17(11):e0276888. doi: 10.1371/journal.pone.0276888 (PMC9632833; doi:10.1371/journal.pone.0276888)
Supplement: Attachment — Submitted filename: Reviewers comments.pdf [file pone.0276888.s004.pdf]

Reviewers comments

The search for novel materials with high efficacy to remove metals from effluents is a topic of great environmental and industrial interest. The manuscript by Ali et al synthesized and characterized $SnCaAl_2O_3$, on which adsorption characteristics Zn^{2+}, Cd^{2+}, and Pb^{2+} were tested and analyzed through batch mode operation. The effects of operating parameters such as adsorbent dosage, initial concentration, pH, and temperature on the removal of Zn^{2+}, Cd^{2+}, and Pb^{2} were investigated. The manuscript could be acceptable for publication in PLOS ONE considering the following comments before a final decision:

1. Acid-based titration (Boehm's titration) method is used to determine the number of surface oxygen groups (acid or basic) present on the carbon surface. Can the authors justify why they did not conduct Boehm's titration?

2. It's recommended to enrich the introduction section by especially indicating the significance of choosing Zn^{2+}, Cd^{2+}, and Pb^{2+} as mode pollutants (mode adsorbate). The reasons lying behind choosing Zn^{2+}, Cd^{2+}, and Pb^{2+} should be clearly presented.

3. More explanation about the data presented in table 4 must be inserted into the text carefully evaluating and comparing the results of the previous studies with the current ones. The author must denote what is natural Ph in table 4 so that a sound comparison with the literature data can be performed.

4. The standard deviation should be included in all numerical results, as error bars (in all Figures) or as +/- sd (text and Tables).

5. The authors should decide whether to put the graph or table for the isotherm. Putting both is redundant. One of them should be moved to the supporting information.

6. There are too many figures, and the author should combine some of them or transfer some of them to SI.

7. I am also wondering in the regeneration, how the separation of the $SnCaAl_2O_3$ was done from the water. Filtration? Centrifugation? Please clarify.

8. It's necessary for the author to give the maximum acceptable limits for Zn^{2+}, Cd^{2+}, and Pb^{2+} respectively in both drinking as well as in wastewater. This can be added in the introductory section.

9. Please address novelty and originality in the introduction and discussion. The acceptance of the manuscript is contingent upon the incorporation of this point. Finally, there is some grammatical error in the manuscript, I strongly recommend that the language should be improved.
