# Supplementary material for: Synthetization and characterization of SnCaAl2O3 nanocomposite and using as a superior adsorbent for Pb, Zn, and Cd ions in polluted water
Source: PLoS One. 2022 Nov 3;17(11):e0276888. doi: 10.1371/journal.pone.0276888 (PMC9632833; doi:10.1371/journal.pone.0276888)
Supplement: Attachment — Submitted filename: Responses to Reviewers #1, 3 PLos-1.docx [file pone.0276888.s005.docx]

**Manuscript ID:** ***PONE-D-22-24872***

**TITLE: *Synthetization and Characterization of SnCaAl_2_O_3_ Nanocomposite as a Superior Adsorbent for Pb, Zn, and Cd Ions in Polluted Water***

***PLOS ONE***

**REVIEWER REPORT(S):**

**Referee:**

**Dear reviewer,**

**Thanks for your comments.**

We have made all the necessary responses to the reviewers' comments; please find them below. All authors have read, approved, and made substantial contributions to this version of the manuscript. Please find, in the following pages, our replies and changes made in the manuscript in response to the reviewers' comments.

**Responses to Reviewer#1**

Generally, thank you for all your constructive comments on this manuscript, which add to my knowledge.

| No. | Reviewer Comments | Responses to reviewer |
| --- | --- | --- |
| 1 | ‎‎ The title can be better and more attractive; it should be more precise and represent to the ‎contents, ‎  ‎ | It has been modified. |
| 2 | ‎  ‎‎ Units in all sections should be uniform, Significant figures should be uniform,‎  ‎ | They have been adjusted. |
| 3 | References must be cited in the correct place in the text, and also must match correctly their ‎position in the list. Please Cite references at appropriate locations and list them correctly. Spell of ‎references must be checked.‎  ‎ | All references have been cited in the correct place in the text. |
| 4 | Ensure that all figures are cited in the text.‎  ‎ | They have been checked. |
| 5 | The abstract is concise and accurately summarizes the essential information. The abstract should be ‎rewritten to summarize the work; the abstract should briefly state the purpose of the ‎research, the principal results, and major conclusions. An abstract is often presented ‎separately from the article, so it must be able to stand alone. Add quantitative data ‎  ‎ | It was highlighted in the abstract section (green highlighted). |
| 6 | More elaboration on the chemical interaction between the components is required.‎  ‎‎ | The current studies covered the chemical interactions required to achieve the present study objective and will be addressed more chemical interactions in future studies. |
| 7 | The introduction; what is the gab to cover  ‎ | It was highlighted in Row 55 to 57 (green) |
| 8 | Batch adsorption study; please add conditions in quantity how the tests were ‎performed | The SnCaAl_2_O_3_ adsorbent dosage effect on Zn^+2^, Cd^+2^, and Pb^+2^ removal via adding various weights ranging from 0.02 up to 1 g to 50 mL (aqueous solution) of 500 mg/L Zn^+2^, Cd^+2^, and Pb^+2^ as an initial concentration, was investigated. |
| 9 | The effect of solution pH is questionable? How come the range of solution pH can be extended up to 9, how about the effect of metal ion precipitation in a basic pH environment? | Dear reviewer, thanks for that comment.  The ppt pH value for each metal ion is considered, and the 9 has the average least ppt of each metal ion under study, so we deleted the values for the figure, as you can see. |
| 10 | Line 257 "its maximum at pH 8 with the highest removal percent of 92.8%, 86%, and 82% for the ions of…" The highest removal at pH 8 is mainly due to the precipitation effect. | The values are removed from the figure, and the removal efficacy is corrected to pH 7, with the highest removal percent of 84.4%, 87%, and 84.8% for the ions of Zn, Pb, and Cd, respectively. |
| 11 | Fig. 9 is questionable, first, the range of solution pH should not exceed 7, second the Y-axis scale for R% should be in range 0-100%.  ‎‎ | Fig. 9 It has been corrected. |
| 12 | Please improve the analysis and interpretation of Adsorption results; the following references are useful: DOI: 10.1007/s10924-021-02160-z; DOI: 10.5004/dwt.2018.21976; DOI: 10.1007/s10924-020-01734-7, DOI: 10.1016/j.ijbiomac.2021.08.160. (ref.) 123;132) | They have been added. |
| 13 | Clearly indicate by numbers how many tests you did under testing: and justify why you ‎selected this  ‎ | The number of tests is three times, and three times were selected, so the results are more accurate.‎ |
| 14 | Captions of the figure and tables must be with complete information, conditions, etc ‎  ‎ | All have been done. |
| 15 | Values in the tables should be of uniform significant figures, please recheck  ‎ | All have been adjusted. |
| 16 | Please improve the conclusion with clear quantitative findings ‎  ‎ | Done, and it was highlighted in the conclusion section |
| 17 | ‎ More emphasis on finding and its implication may be mentioned in the conclusion section.‎  ‎ | The conclusion has been modified. (green highlighted) |
| 18 | Typos are to be corrected, also check the equations, English must be improved .‎  ‎ | All typos, equations, and grammar have been improved. |
| 19 | Add experimental conditions to the captions of each figure.‎ | They have been added. |

**Responses to Reviewer #2**

Generally, thank you for all your constructive comments on this manuscript, which add to my knowledge.

| No. | Reviewer Comments | Responses to reviewer |
| --- | --- | --- |
| 1 | Acid-based titration (Boehm’s titration) method is used to determine the number of surface oxygen groups (acid or basic) present on the carbon surface. Can the authors justify why they did not conduct Boehm’s titration? | This method is suitable for acidic and basic materials that carry these moving ions on their surface. Still, the prepared compound carries the charge as a result of ions acquired on the surface, so this measurement is done in a newer way, using zeta potential, which is an accurate method and gives precise results for the surface charge of the prepared materials. |
| 2 | It’s recommended to enrich the introduction section by especially indicating the significance of choosing Zn^2+^, Cd^2+^, and Pb^2+^ as mode pollutants (mode adsorbate). The reasons lying behind choosing Zn^2+^, Cd^2+^, and Pb^2+^ should be clearly presented. | Done, and it was highlighted in Row 45 |
| 3 | More explanation about the data presented in table 4 must be inserted into the text carefully evaluating and comparing the results of the previous studies with the current ones. The author must denote what is natural pH in table 4 so that a sound comparison with the literature data can be performed. | Done, and it was highlighted in Row 470 |
| 4 | The standard deviation should be included in all numerical results, as error bars (in all Figures) or as +/- sd (text and Tables). | The standard error of the mean has been added to the column figures due to the excellent presentation. |
| 5 | The authors should decide whether to put the graph or table for the isotherm. Putting both is redundant. One of them should be moved to the supporting information. | Done, the figure was removed |
| 6 | There are too many figures, and the author should combine some of them or transfer some of them to SI. | The figures of kinetic and isotherm models have been transferred to the supplementary file (S1). |
| 7 | I am also wondering, in the regeneration, how the separation of the SnCaAl_2_O_3_ was done from the water. Filtration? Centrifugation? Please clarify. | The separation of the prepared SnCaAl_2_O_3_ was done from the water by Filtration and recycled. |
| 8 | It’s necessary for the author to give the maximum acceptable limits for Zn^2+^, Cd^2+^, and Pb^2+^, respectively, in both drinking as well as in wastewater. This can be added in the introductory section. | Done, and it was highlighted in Row 46 to 48 |
| 9 | Please address novelty and originality in the introduction and discussion. The acceptance of the manuscript is contingent upon the incorporation of this point. | Done, and it was highlighted in the introduction in Row 66 to 70 and discussion in Row 128 to 131 |
| 10 | Finally, there is some grammatical error in the manuscript; I strongly recommend that the language should be improved. | It has been corrected in the whole manuscript. |

**Responses to Reviewer#3**

| No. | Reviewer Comments | Responses to reviewer |
| --- | --- | --- |
| 1 | Abstract- "Moreover, the adsorption thermodynamic behavior of Zn+2, Cd+2, and Pb+2 30 on the synthesized composite." – The metal ions should be written uniformly with valency state throughout the manuscript. | It has been done. |
| 2 | "Water pollution by toxic heavy metal ions is a critical environmental problem that may pose serious health effects." --- I suggest the author discuss a paragraph related to water pollution due to the presence of different contaminants and applications of different adsorbents for the treatment techniques. The authors are recommended to check the below related references, which will improve the supporting information.  Journal of Cleaner Production 241, 2019, 118263  Environmental research 2019, 170, 389-397  Journal of Molecular Liquids 317, 2020, 113916  Journal of environmental management 219, 2018, 285-293 | Done, and it was highlighted in Rows 42:43, and 50 to 57. |
| 3 | The author mention, "Adsorption of ions on the surface of many solid materials such as clay, zeolites, activated charcoal, or silica gel is the common removal system" – The statement needs supporting citations as well.  Journal of Hazardous Materials 400, 2020, 123247  Desalination and Water Treatment 57 (46), 2016, 21863-21869  Nanomaterials 9 (5), 2019, 776  Microporous and Mesoporous Materials 261, 2018, 198-206 | Done, and it was highlighted in Row 55 to 57 |
| 4 | "A known amount of the as-prepared nano Al2O3 shells was first dispersed in 20 mL of deionized water, followed by a pre-determined amount of CTAB, CaCl2, and SnCl2 with stirring and heating at 300 rpm at 40 °C for 2 h, respectively"-Does the author followed any reported literature? | Yes, It followed the literature, and references have cited it.  1. Jong-Hwan Yoon (2019) "Fabrication of Sn@Al_2_O_3_ Core-shell Nanoparticles for Stable Nonvolatile Memory Applications" Materials, MDPI  Raw 80  2. Sidra Hashmi , Sumbal Gohar , Tariq Mahmood, Umar Nawaz4 , Hadayatullah Farooqi( 2016) "Biodiesel Production by using CaO-Al_2_O_3_ Nano Catalyst" International Journal of Engineering Research & Science (IJOER) ISSN: [2395-6992] [Vol-2, Issue-3 in Raw83 |
| 5 | The author need to discuss the regeneration methods of the adsorbent materials. | The regeneration process has been discussed. |
| 6 | The English quality not up to the mark. All the typos and grammar need to check thoroughly in the manuscript. | The English quality has been enhanced thoroughly in the manuscript. |
| 7 | "Figure 10. The dose effect on the SnCaAl2O3 nanoparticles adsorptions."—Add the optimal conditions in each caption. | It was added and highlighted in the captions of each figure. |

Thank you very much

For your cooperation

Corresponding author

***Abeer El Shahawy***
